# Differential association of CD68^+^ and CD163^+^ macrophages with macrophage enzymes, whole tumour gene expression and overall survival in advanced melanoma

**DOI:** 10.1038/s41416-020-01037-7

**Published:** 2020-08-26

**Authors:** Liam Friel Tremble, Mark McCabe, Sidney P. Walker, Siobhán McCarthy, Réiltín F. Tynan, Suzanne Beecher, Réiltín Werner, A. James P. Clover, X. Derek G. Power, Patrick F. Forde, Cynthia C. B. B. Heffron

**Affiliations:** 1 Cancer Research @ UCC, University College Cork, Cork, Ireland; 2 Department of Pathology, Cork University Hospital, Cork, Ireland; 3 APC microbiome Ireland, University College Cork, Cork, Ireland; 4 Department of Dermatology, South Infirmary Victoria University Hospital, Cork, Ireland; 5 National University of Ireland, Galway, Ireland; 6 Cork University Hospital, Cork, Ireland; 7 Department of Medical Oncology, Mercy and Cork University Hospitals, Cork, Ireland

**Keywords:** Tumour immunology, Tumour immunology

## Abstract

**Background:**

The density and phenotype of tumour-associated macrophages have been linked with prognosis in a range of solid tumours. While there is strong preclinical evidence that tumour-associated macrophages promote aspects of tumour progression, it can be challenging to infer clinical activity from surface markers and ex vivo behaviour. We investigated the association of macrophage infiltration with prognosis and functional changes in the tumour microenvironment in primary human melanoma.

**Methods:**

Fifty-seven formalin-fixed, paraffin-embedded primary melanomas were analysed by immunohistochemical analysis of CD68, CD163, inducible nitric oxide synthase (iNOS) and arginase expression. RNA sequencing was performed on serial sections of 20 of the stained tumours to determine the influence of macrophage infiltration on gene expression.

**Results:**

CD68^+^ cells are a functionally active subset of macrophages that are associated with increased iNOS and arginase staining and altered gene expression. In comparison, while there is a greater accumulation of CD163^+^ macrophages in larger tumours, these cells are comparatively inactive, with no association with the level of iNOS or arginase staining, and no effect on gene expression within the tumour. The infiltration of either subset of macrophages did not correlate to overall survival.

**Conclusions:**

Thus, melanomas contain distinct macrophage populations with diverse phenotypes, but with no observable prognostic role.

## Background

Macrophage infiltration, as determined by the evaluation of FFPE (formalin-fixed paraffin-embedded) tissues, has been shown to be a prognostic indicator in a range of solid malignancies.^1^ The markers used to identify macrophages vary, and there is no consensus on markers that reflect true physiological subsets.

In the majority of cancers, the macrophage marker CD163 correlates with more advanced tumours and to a worse prognostic outcome, except in the cases of gastric cancer and colorectal cancers.^1–4^ Other markers, such as CD68, have been used to stain macrophages, but have been linked to both favourable and unfavourable outcomes.^4,5^

Previous studies have attempted to delineate the prognostic influence of macrophage infiltration in melanoma. While some studies have observed no prognostic role, in others both the number of CD68^+^ cells and soluble CD163 have correlated to poor overall survival (OS); however, only CD68^+^ cell counts at the invasive front of the tumour have been found to be independent predictors of reduced survival.^6–9^

Macrophages have been implicated in all aspects of tumour progression, including proliferation, survival, immunosuppression, angiogenesis and metastasis.^10^ Furthermore, macrophages have been implicated in resistance to various therapies, such as chemotherapy and radiotherapy.^11–14^

However, much evidence of the effects of intratumoural infiltration have been inferred from in vitro analysis, which is commonly based on the hypothesis that the expression of surface markers is reflective of functional phenotype; however, it is known that the diversity of functional phenotypes and macrophage plasticity are controlled by a much more intricate underlying system of epigenetics that cannot be represented by the known subset of surface markers.^15^ The precise nature of macrophages continues to be unravelled, and during the process of unravelling, further findings continue to add additional layers of complexity to an already poorly understood science. Recently, a horizontal transformation from macrophage-like cells to fibroblast-like cells has been observed, and the source and stability of discrete populations are still not known.^16^ As such, macrophage markers may not be considered stable markers of distinct cells. However, whether they represent stable populations or a continuous flow of cells through a transition state, we can use these markers to identify distinct subsets of macrophage-like cells at any point in time.

Another source of concern is the discrepancy of results in murine and human interventional studies. It is known that monocyte and macrophage markers vary between mice and humans, such as F4/80, which is a monocyte and macrophage marker in mice, but its homologue, EMR1, is an eosinophil-specific receptor in humans.^17^ However, cross-species differences have also been seen in the outcomes of interventions targeting conserved targets. Anti-CSF1R antibodies that inhibit macrophage migration to the tumour have been effective in the treatment of murine malignancies; however, clinical trials have observed no therapeutic benefit in humans, indicating a differential physiological role or reliance on macrophage behaviour between mice and human disease.^1,18,19^ The influence of macrophages on tumour biology in human malignancies is not fully known as macrophages are also known to adopt unreactive senescent and quiescent states.^20^

Another central concern is the sensitivity of macrophages to environmental stimuli, which adds a question to the compatibility of ex vivo studies. Macrophages are known to be activated by environmental stimuli, including adherence to the extracellular matrix and a wide range of innocuous stimuli such as adherence to plastic; thus, ex vivo studies contain inherent limitations.^21,22^ An alternative approach is to investigate the presence and associations of macrophages in formalin-fixed paraffin-embedded (FFPE) tissues. While this approach reduces the level of artificially introduced environmental impacts, it comes with its own experimental limitations. From a clinical standpoint, this approach has significant advantages in being able to draw on the existing biobanks with associated patient data.

Here, we evaluate the presence of CD68^+^ and CD163^+^ macrophages in FFPE human primary melanoma lesions and determine their correlation with the functional canonical M1:M2 enzymes inducible nitric oxide synthase (iNOS) and arginase. To further investigate if these cells have a biological role, we investigate their link with OS and determine if they correlate to differential gene expression within the tumour.

## Methods

### Patient cohort

Fifty-seven primary tumour blocks were randomly selected from BRAF-tested tumours; for each tumour, a single block with a large area of both tumour and peritumoural tissue was selected. Patients were untreated prior to tumour excision. Pathological data and follow-up data were obtained from patient charts. Tumour and cohort descriptions are provided (Supplementary Tables 1, 2).

### Immunohistochemistry

Serial 3 μM sections were cut and mounted on Superfrost Plus slides (Thermo Scientific). Unstained slides were kept at 4 °C and stained for a 1 month. Slides were heated at 37 °C overnight or 60 °C for 60 min to begin antigen retrieval.

iNOS staining was performed on the bench in a humidified chamber where appropriate. Slides were briefly boiled in citrate buffer pH 6 for 20 min and placed in 3% hydrogen peroxide for 10 min. Slides were washed, which entailed gentle serial immersions in three phosphate-buffered saline (PBS) baths for 5 min each. Slides were blocked with 5 mg/ml bovine serum albumin in PBS for 1 h. iNOS antibody (SP126, Invitrogen) was diluted 1 in 100 in a blocking buffer, and the slides were covered in antibody solution for 60 min. Slides were washed and immersed in secondary antibody (goat anti-rabbit IgG heavy and light chain (H&L) alkaline phosphatase, Abcam ab97048, 1 in 1000 in blocking buffer) for 30 min. Slides were washed and covered in Fast-Red for 30 min. Slides were rinsed in water, counterstained in Mayer’s haematoxylin and blued in Scott’s tap water. Coverslips were mounted on an aqueous mounting medium.

CD68 (PA0273, Leica Biosystems), CD163 (MRQ-26, Merck) and arginase (Sigma) staining was performed on an automated Benchmark Ultra (Roche) using the ultraView Universal Alkaline Phosphatase Red Detection Kit (Roche). In brief, slides were deparaffinised, and antigen retrieval was performed in Cell Conditioning 1 (Ventana Medical Systems, Inc.) for 20 min (arginase), 64 min (CD163) or 20 min (CD68) before the addition of one drop of primary antibody for 60 min (arginase) or 32 min (CD163 and CD68). Slides were counterstained in haematoxylin II, blued in bluing reagent and coverslips were mounted in the aqueous mounting medium.

Stained slides were imaged and quantitatively measured in three peritumoural and three intratumoural areas of highest staining density. Positive cells per mm^2^ were counted by two trained pathologists (C.H. and M.M.) and averaged. Due to a high number of specimens that were negative for CD68^+^ and iNOS^+^ cells in the tumoural and peritumoural tissue, CD68 and iNOS staining was stratified semi-quantitatively as positive (containing one or more positive cells) or negatively stained (no positive cells). All analyses were also performed with continuous data to ensure the presentation of biologically relevant data.

CD68, iNOS and arginase specifically stained cytoplasmic compartments (Supplementary Fig. 1). CD163 stained both the cytoplasm and plasma membrane of target cells, with increased staining intensity seen at the plasma membrane. CD68 and iNOS staining was specific to cells with a distinct macrophage morphology. CD163 stained a much larger number of cells, but was not restricted to cells of a distinct macrophage-like morphology. Arginase staining was less frequent and was completely negative in almost half of stained sections. In positive sections, stained cells showed a distinct macrophage (Supplementary Fig. 1e) or neutrophil (Supplementary Fig. 1f) morphology; thus, arginase staining, as presented in this study, is a reflection of a subset of both neutrophils and macrophages. Many ulcerated tumours showed a distinct influx of a high number of arginase^+^ neutrophils to the peritumoural ulcerated tissue. There was a statistically significant positive correlation between peritumoural arginase^+^ cells and ulceration, accompanied by a significant decrease in peritumoural CD68^+^ cell infiltration.

### RNA sequencing

RNA sequencing was performed on 5 μM sections mounted on Superfrost Plus slides. Serial sections were used to stain a haematoxylin and eosin (H&E) slide, from which tumour tissue was outlined. Using the H&E as a guide, tumour tissue was lysed in situ on the slide and RNA sequencing was performed using HTG EdgeSeq technology with the HTG EdgeSeq Oncology Biomarker Panel. Pathological features of the 20 tumours selected for gene expression analysis are shown (Supplementary Table 2).

### Statistical analysis

Spearman’s correlations were used to determine significance and determine correlation coefficients between continuous variables. The Wilcoxon rank-sum test was used to determine significance between continuous and discrete variables.

**Fig. 1:**
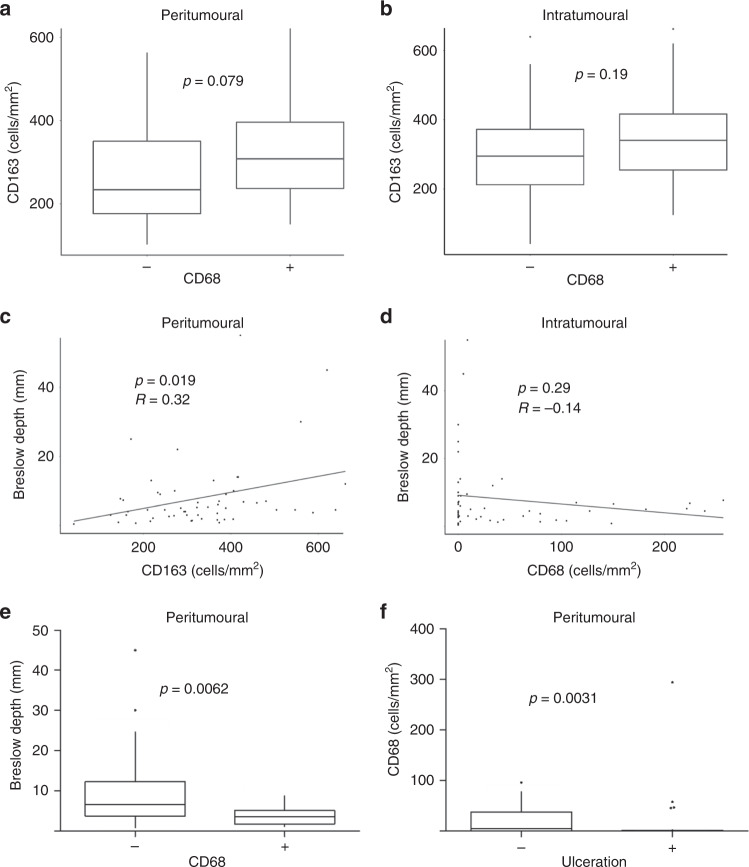
Differential CD68^+^ and CD163^+^ macrophage recruitment to primary melanoma lesions. The number of CD68^+^ and CD163^+^ cells was determined by immunohistochemistry in primary melanoma tumours. The number of positive cells was counted in the peritumoural and intratumoural regions. CD68^+^ and CD163^+^ cells were shown to be differentially recruited (**a**, **b**). Intratumoural CD163^+^ macrophage infiltration was positively correlated to Breslow depth (**c**), while intratumoural CD68^+^ macrophage infiltration showed no significant correlation (**d**). Tumours with no peritumoural CD68^+^ macrophages had a greater Breslow depth (**e**), and decreased numbers of peritumoural CD68^+^ macrophages were seen in ulcerated tumours (**f**).

Where a large number of zero values were obtained in immunohistochemical measurements (CD68 staining), semi-quantitative measurements were used for data analysis. A Spearman’s correlation was used in Fig. 1d to show the association of intratumoural CD68^+^ cells with Breslow depth; however, the Wilcoxon rank-sum test value of this association is also presented in Supplementary Table 3. Both were non-significant.

All statistical analyses were carried out in the R environment, v.3.4.4. Boxplots shown represent the mean, with interquartile ranges (boxes) and 95% confidence interval (whiskers), all outliers are shown.

Gene expression data were analysed using normalised gene values as presented by HTG EdgeSeq technology. Differentially expressed genes between groups were detected using the DESeq2 algorithm with adjusted *p* values. Heat maps were clustered using the Ward-Linkage method and generated using the Made4 package v.1.58 within the R environment. Kaplan–Meier plots and associated statistical testing was performed using the survminer package in R, v.0.4.4. Survival probabilities were calculated using the Cox proportional hazards model, and differences between groups were analysed using the log-rank test.

## Results

### Correlation of CD68^+^ and CD163^+^ macrophage infiltration with pathological features of melanoma

Primary untreated melanoma lesions were stained with CD68 and CD163 antibodies to identify macrophage populations, and the number of positive cells per mm^2^ was measured in both intratumoural tissue and peritumoural tissue. Twenty-one specimens were negative for intratumoural CD68^+^ cells and 30 were negative for peritumoural CD68^+^ cells, as such CD68 data were stratified into those with and without the presence of stained cells. Previous reports have similarly reported that the presence of CD68^+^ cells could be a negative or a positive prognostic marker across tumours of the same subtype.^23^

There was no significant correlation between the infiltration of CD163^+^ and CD68^+^ cells, indicating that these cells represent distinct, but potentially non-mutually exclusive, macrophage subsets (Fig. 1a, b). The correlation of CD68^+^ and CD163^+^ macrophage infiltration with pathological features of the tumour was determined using Spearman’s correlations and Wilcoxon rank-sum tests (Supplementary Table 3). No differences in cell density were seen between baseline characteristics, such as age, gender, tumour stage or tumour-infiltrating lymphocytes. Intratumoural CD163^+^ macrophages were found to increase with Breslow depth (Fig. 1c). There was a significant decrease in the number of peritumoural CD68^+^ macrophages in tumours with a thicker Breslow depth (Fig. 1e); however, there was no correlation with intratumoural CD68^+^ macrophages and tumour size (Fig. 1d). A reduced number of peritumoural CD68^+^ cells were also seen in ulcerated tumours (Fig. 1f).

### Correlation of CD68^+^ and CD163^+^ macrophage infiltration with the number of iNOS^+^ and arginase^+^ cells

**Fig. 2:**
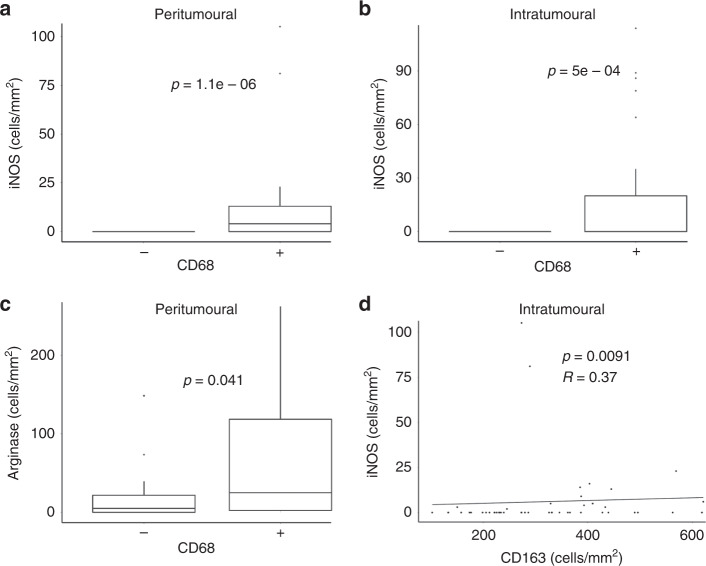
Association of CD68^+^ and CD163+ macrophage infiltration with iNOS^+^ and arginase^+^ cells. The number of CD68^+^, CD163^+^, iNOS^+^ and arginase^+^ cells was determined by immunohistochemistry in primary melanoma tumours. The number of positive cells was counted in the peritumoural and intratumoural regions. **a**, **b** Intratumoural CD68^+^ macrophages were positively correlated to the number of iNOS^+^ arginase^+^ cells. **c** Peritumoural CD68^+^ macrophages were found to positively correlate to arginase^+^ cells. **d** Intratumoural CD163^+^ macrophages were found to correlate to the number of iNOS^+^ cells; however, the trend line shows that this is a near-zero trend.

Given that CD68^+^ and CD163^+^ macrophages represent distinct subsets with distinct staining profiles based on a number of pathological features, we next determine if these differences reflected different immunological profiles within the tumour by measuring the expression of the macrophage enzymes iNOS and arginase. These enzymes were selected as they are constitutively active when expressed; thus, they are likely to have a meaningful impact on tumour biology, and they are highly cited in the literature. However, it must be stated that while these enzymes are associated with inflammatory or tissue repair phenotypes, respectively, it is important not to infer a distinct M1 or M2 phenotype in these cells. Strong positive correlations were seen between intratumoural CD68^+^ macrophages and the number of both iNOS^+^ and arginase^+^ cells. Specimens with intratumoural CD68^+^ macrophages had a higher level of both arginase^+^ and iNOS^+^ cells (Fig. 2a, b), while specimens with peritumoural CD68^+^ macrophages also showed a strong increase in peritumoural arginase levels (Fig. 2c). In contrast, there were no strong positive or negative correlations between CD163^+^ macrophage density and the number of either iNOS^+^ or arginase^+^ cells. Intratumoural CD163^+^ macrophage density was significantly correlated to the number of iNOS^+^ cells showing no meaningful change in the number of iNOS^+^ cells, regardless of CD163^+^ macrophage density (Fig. 2d). Thus, strong differences exist between the correlations of CD68^+^ and CD163^+^ macrophage populations and iNOS or arginase activity.

### Effect of CD68^+^ and CD163^+^ macrophage infiltration on total intratumoural gene expression

**Fig. 3:**
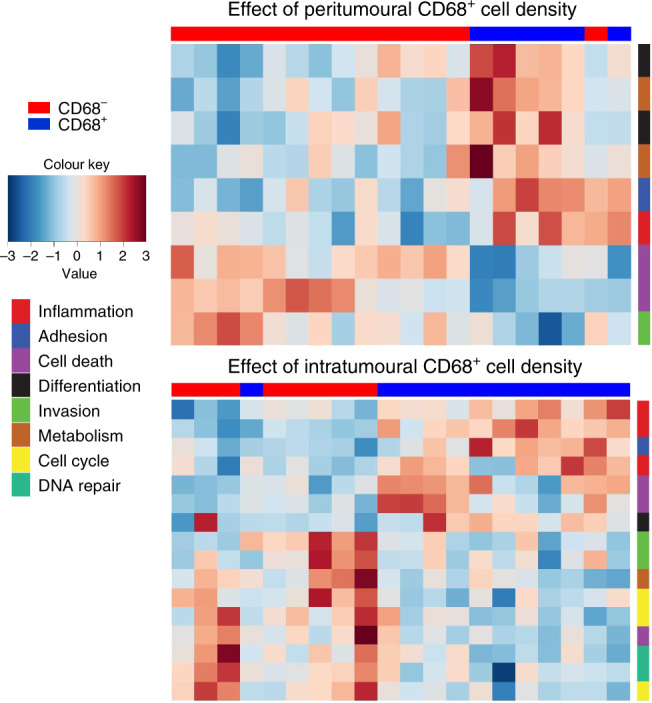
The correlation of CD68^+^ macrophage recruitment with differential gene expression. The number of CD68^+^ and CD163^+^ macrophages was determined by immunohistochemistry in primary melanoma tumours. Intratumoural gene expression of the same FFPE blocks was measured using next-generation sequencing technology. Using Deseq2 algorithms, both peritumoural and intratumoural CD68^+^ macrophages were shown to correlate to differential regulation of a number of genes within the tumour. CD68^+^ samples are marked as blue along the upper margin, while CD68^−^ samples are marked as red. Gene-associated signalling pathways are colour coded on the right-hand margin. The level of CD163^+^ macrophage infiltration observed no effect.

While an increase in the number of iNOS^+^ and arginase^+^ cells in tumours with CD68^+^ macrophages is indicative of an active CD68^+^ macrophage phenotype, both iNOS and arginase activity reflects a very small component of macrophage behaviour, which is unlikely to determine the influence of macrophages on tumour biology independently. To determine if the macrophage infiltration could have an effect on global gene expression within the tumour tissue, we performed RNA sequencing on serial sections of the same FFPE tissue. In addition to seeing no effect of CD163^+^ macrophage infiltration on iNOS or arginase staining, no effect was seen on gene expression within the tumour tissue. Analysis of both peritumoural and intratumoural CD68^+^ macrophage infiltration correlated to differential expression of a number of genes within the tumour tissue (Fig. 3). This effect was all the more notable given an average of 7–15-fold more CD163^+^ macrophages in the tumour in comparison to CD68^+^ macrophages. The genes affected were not specific to monocytic cells and were reflective of a broad range of processes within the tumour, such as cell proliferation, cell death and differentiation.

### Effect of CD68^+^ and CD163^+^ macrophage infiltration on OS

**Fig. 4:**
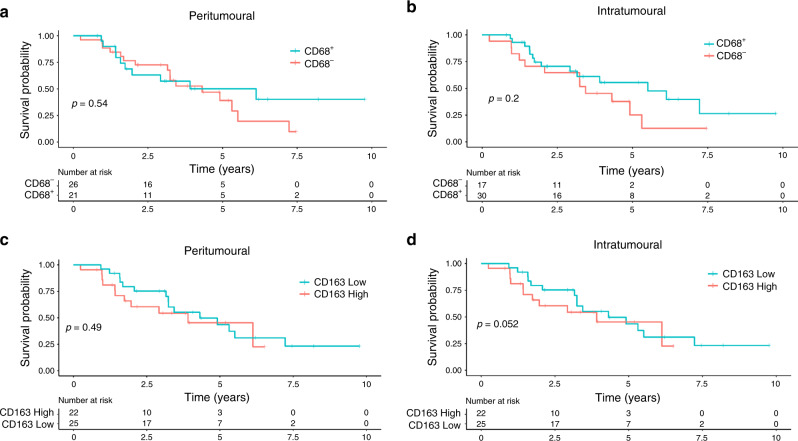
Correlation of CD68^+^ and CD163^+^ macrophage infiltration with OS. The number of peritumoural and intratumoural CD68^+^ and CD163^+^ macrophages was determined by immunohistochemistry in primary melanoma tumours. Patients were stratified based on the density of infiltrating macrophages, and survival probabilities were predicted using Cox proportional hazards model and visualised with a Kaplan–Meier curve. Tumours were stratified as having CD68^+^ macrophages (positive) or having no CD68^+^ cells present (negative) (**a**, **b**). The level of CD163^+^ infiltration was stratified as high or low (**c**, **d**).

To determine if the markedly different functional phenotypes of CD68^+^ and CD163^+^ could impact the patient outcome, we analysed the effect of macrophage density on OS (Fig. 4). No significant correlations were seen in survival probability between tumours with or without CD68^+^ macrophage infiltration or high or low CD163^+^ macrophage infiltration (Fig. 4a–d).

Previous studies have shown contrasting prognostic trends of CD68 M1-like and CD163 M2-like macrophages in other tumour types, such as lung cancer and ovarian cancer.^24,25^ Non-significant trends of increased intratumoural CD68^+^ (*p* = 0.2) and CD163^+^ (*p* = 0.052) macrophage density were associated with improved and worse survival, respectively. However, these results are in line with previous literature, which have failed to show a prognostic significance of tumour-associated macrophage (TAM) density in melanoma.

### Correlation of iNOS^+^ and arginase^+^ cell infiltration with pathological features of melanoma

**Fig. 5:**
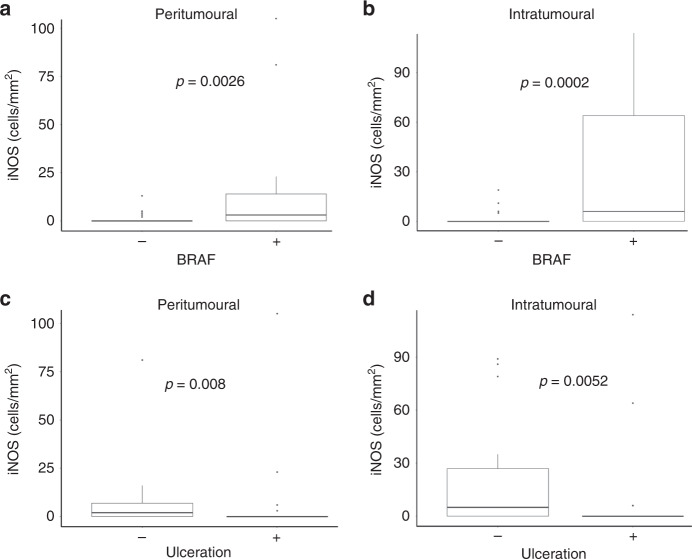
Correlation of iNOS^+^ cell density with ulceration and BRAF status. The number of peritumoural and intratumoural iNOS^+^ cells was determined by immunohistochemistry in primary melanoma tumours. Significant correlations of iNOS^+^ cell density with the pathological features BRAF status (**a**, **b**) and ulceration (**c**, **d**) are shown.

Having shown the effect of CD68^+^ and CD163^+^ macrophage infiltration, we next observed if the number of iNOS^+^ or arginase^+^ cells independently correlated to pathological features of the tumour (Supplementary Table 4). There were no correlations with age, gender, Breslow depth, tumour stage, mitotic count or tumour-infiltrating lymphocytes. BRAF^+^ tumours were found to have significantly increased levels of peritumoural and intratumoural iNOS^+^ cells (Fig. 5a, b). Ulceration and BRAF status correlated to the number of iNOS^+^ and arginase^+^ cells. Notably, the presence of ulceration resulted in significantly reduced peritumoural and intratumoural iNOS^+^ cells (Fig. 5c, d).

### Correlation of BRAF mutational status with CD68^+^ macrophage recruitment and total intratumoural gene expression

**Fig. 6:**
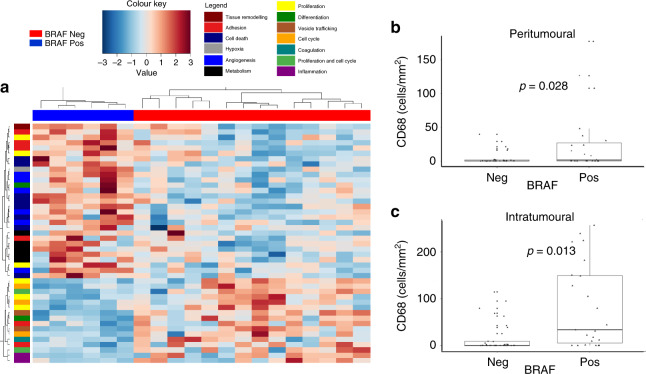
The effect of BRAF status on CD68^+^ macrophage density and gene expression. The number of CD68^+^ macrophages was determined by immunohistochemistry in primary melanoma tumours. **a** Intratumoural gene expression of the same FFPE blocks was measured using next-generation sequencing technology. The BRAF status of samples is shown along the upper margin with BRAF mutation-positive samples marked blue and BRAF mutation-negative samples marked red. Using Deseq2, genes differentially expressed based on BRAF status were identified. Gene-associated signalling pathways are colour coded on the left-hand margin. Using Wilcoxon rank-sum tests, it was shown that there were a higher number of peritumoural and intratumoural CD68^+^ macrophages in BRAF^+^ tumours (**b**, **c**).

In addition to the positive correlations of CD68^+^ macrophages with iNOS^+^ cells and iNOS^+^ cells with BRAF status, we found that there were a significantly higher number of CD68^+^ macrophages in BRAF^+^ melanomas (Fig. 6b, c). BRAF status had significant effects on gene expression within the tumour (Fig. 6a). The genes affected by BRAF were independent of the subset affected by CD68^+^ macrophage infiltration, with the exception of VCAM1 and CDKN3, indicating distinct physiological regulation by CD68^+^ infiltration and BRAF positivity. BRAF^+^ tumours had upregulated genes involved in angiogenesis, cell death and metabolism, but down-regulated genes associated with proliferation, cell cycle and inflammation.

## Discussion

Previous reports have cited CD68 as a pan-macrophage marker and CD163 as an M2-like macrophage marker.^26^ Staining of primary melanoma specimens has shown that CD68^+^ cells are significantly less prevalent than CD163^+^ cells. In many melanomas, there were no CD68^+^ macrophages within tumours or peritumoural tissue, but there were high levels of CD163^+^ macrophages across almost all cases. Thus, it is clear that CD68 is not a pan-macrophage marker in melanoma.

CD68^+^ and CD163^+^ macrophages represent distinct physiological subsets. While M2 macrophages are commonly regarded as promoting disease progression, we saw no evidence of a biological role of these cells, except an accumulation of cells during disease progression, as seen by the positive correlation between intratumoural CD163^+^ macrophages and Breslow depth. Despite the high numbers of these cells (up to 1161 positive cells per mm^2^), there was no correlation with an increase in iNOS^+^ or arginase^+^ cells, implying that they do not align with traditional M1:M2 phenotyping. Furthermore, they had no significant effect on gene expression within the tumour, indicating that a high number of these cells may not physiologically impact tumour biology. While there was a non-significant correlation with OS, it is possible that this trend is reflective of the increased Breslow depth of tumours with a high number of CD163^+^ cells, and not an independent negative prognostic effect of the cells themselves.^27^

Our study cohort was selected from patients who underwent routine BRAF status testing and was thus skewed towards advanced melanoma patients. Therefore, the lack of correlation of macrophage markers with tumour stage cannot be meaningfully interpreted and will require analysis in an appropriately powered cohort. Previous investigations have suggested there is an increase in the density of CD68^+^ macrophages in advanced tumour stages.^28^

The contrasting trends of CD68^+^ macrophages and CD163^+^ macrophages against Breslow depth is striking in suggesting a differential expression pattern, albeit with the former being non-significantly correlated. A recent study by Lee et al.^29^ provides corroborating evidence that increased CD163^+^ cell infiltration is associated with increased Breslow depth.^29^ There is much evidence that tumours exert a strong polarising effect on recruited cells, and it remains to be determined whether the CD68^+^ and CD163^+^ infiltration is reflective of differential recruitment, or a system in which CD68^+^ cells are recruited and polarised to a CD163^+^ phenotype, which is less functionally active in the parameters we observed, such as arginase and iNOS activity and influencing gene expression within the tumour.^30–32^ This system would explain both the increased prevalence of CD68^+^ macrophages in less progressed tumours and increased accumulation of CD163^+^ macrophages in more advanced tumours. Previous reports have shown that there is a continuous supply of monocytes to diverse tumour types, even tumours such as pancreatic ductal adenocarcinoma, which has a dense desmoplastic barrier surrounding the tumour tissue that can impair cell infiltration, and that tumours exert a strong polarising effect on TAMs to a more M2-like phenotype.^33–36^

A high density of intratumoural and peritumoural CD163^+^ macrophages were detected in many tumours; however, this failed to translate into a difference in global gene expression within the tumour tissue. Previous reports have shown an association between CD163^+^ macrophage infiltration and angiogenesis and cyclooxygenase-2 expression.^29^ Specific analysis of these genes with unadjusted *p* values, which may not have been detected by DESeq2 analysis of total gene expression data sets, showed no correlation of CD163^+^ macrophage density with the expression of the genes VEGFA/B/C, ANGPT1/2 or PTGS1/2.

The strong positive correlations between CD68^+^ macrophage infiltration and the number of iNOS^+^ cells gives strong reason to suggest that these cells are more M1-like cells. However, the positive correlation with arginase^+^ cells is more surprising, but possibly representative of a more immunologically active subset capable of a range of responses.

This argument is upheld by the effect of CD68^+^ macrophage infiltration on gene expression within the tumour, in which increased infiltration can impact genes involved in a range of processes, such as cell death and cell cycle, in addition to the role of regulation of inflammation.

While no correlation of CD68^+^ or CD163^+^ cell density was found on OS, non-significant trends were seen, which align with previous observations in other tumour types in which CD68^+^ cells can be associated with improved prognosis and CD163^+^ cells can be associated with worse prognosis in a range of solid malignancies.^1^

The increased presence of CD68^+^ cells and inflammatory markers in BRAF^+^ tumours could indicate that BRAF status may play a role in determining the response of patients to immunotherapies or immunogenic treatments. Recently, CD68^+^ macrophages have been found to promote tumour hypoxia and drive resistance to anti-PD-1 antibodies in murine models.^37^ Conversely, M1-like CD68^+^ macrophages were also found to potentiate the synergistic effects of VEGF inhibitors with BRAF inhibitors in murine melanoma.^38^ However, clinically CD68 has been found to be a biomarker of response to ipilimumab.^39^ Melanoma patients with BRAF mutations were found to have longer progression-free survival and higher OS in response to Nivolumab, with or without ipilimumab; thus, CD68^+^ macrophages may potentiate the favourable effect of BRAF status during treatment with immunotherapies.^40^

While the two were positively associated, the different genes affected by BRAF status and the presence of CD68^+^ macrophages indicate non-redundant roles of BRAF status and CD68^+^ cell infiltration. The p53-DREAM pathway genes *CDKN3*, *DEPDC*1, *BIRC5*, *BRIP1*, and *RFC4* were all strongly associated and down-regulated in tumours with intratumoural CD68^+^ macrophage infiltration.^41^ None of these genes was affected by BRAF status, indicating that this pathway is regulated by macrophages.

The highly active functional phenotype of CD68^+^ macrophages may indicate that they are a more viable therapeutic target for therapies looking to boost or interfere with TAM behaviour. Previous clinical interventions have targeted CSF1R due to the increased prevalence of this receptor and the quality of antibodies, which can be raised against this target.^1^ As CSF1R is a G protein-coupled receptor, the opsonisation and depletory effects of targeting are augmented by the inhibition of survival signals in targeted cells. Therapeutic targeting of CD68 cells has been impeded by the discrepancies between human CD68 and its murine homologue, macrosialin, which is not restricted to monocytic cells in mice. However, CD68 has been shown to represent differential macrophage subsets to both CSF1R and CD163, and thus depletion of CD68^+^ macrophages, if cell-specific targeting could be achieved, could improve therapeutic outcomes.^42–44^

While CD68 has frequently been referred to as a specific monocyte and macrophage marker, a major issue with the targeting of CD68 is its wide expression in a number of cell types. Immunohistochemical analysis and RNA sequencing have shown that CD68 levels on fibroblasts and endothelial cells can match levels shown on macrophages, and lower levels can be also be detected on tumour cell lines and lymphoid cells.^42^ This presents major challenges for the effective specific targeting of CD68^+^ macrophages therapeutically. There is merit in investigating if other interventions can be developed, which can boost the activity or the number of CD68^+^ macrophages in the TME. However, these results suggest a hypothesis for the clinical failure of anti-CSF1R antibodies, which do not distinguish between CD68^+^ and CD163^+^ macrophages, and in which the majority of targeted cells are likely to be less functionally active.

## Supplementary information

Supplementary material

## Data Availability

All raw data sets used for analysis can be supplied by the corresponding author.

## References

[1] Tremble LF, Forde PF (2017). Clinical evaluation of macrophages in cancer: role in treatment, modulation and challenges. Cancer Immunol. Immunother..

[2] Forssell J, Oberg A, Henriksson ML, Stenling R, Jung A, Palmqvist R (2007). High macrophage infiltration along the tumor front correlates with improved survival in colon cancer. Clin. Cancer Res..

[3] Zhou Q, Peng RQ, Wu XJ, Xia Q, Hou JH, Ding Y (2010). The density of macrophages in the invasive front is inversely correlated to liver metastasis in colon cancer. J. Transl. Med..

[4] Ohno S, Inagawa H, Dhar DK, Fujii T, Ueda S, Tachibana M (2003). The degree of macrophage infiltration into the cancer cell nest is a significant predictor of survival in gastric cancer patients. Anticancer Res..

[5] Zhang QW, Liu L, Gong CY, Shi HS, Zeng YH, Wang XZ (2012). Prognostic significance of tumor-associated macrophages in solid tumor: a meta-analysis of the literature. PLoS ONE.

[6] Jensen TO, Schmidt H, Moller HJ, Hoyer M, Maniecki MB, Sjoegren P (2009). Macrophage markers in serum and tumor have prognostic impact in American Joint Committee on Cancer stage I/II melanoma. J. Clin. Oncol..

[7] Piras F, Colombari R, Minerba L, Murtas D, Floris C, Maxia C (2005). The predictive value of CD8, CD4, CD68, and human leukocyte antigen-D-related cells in the prognosis of cutaneous malignant melanoma with vertical growth phase. Cancer.

[8] Falleni M, Savi F, Tosi D, Agape E, Cerri A, Moneghini L (2017). M1 and M2 macrophages’ clinicopathological significance in cutaneous melanoma. Melanoma Res..

[9] Salmi S, Siiskonen H, Sironen R, Tyynela-Korhonen K, Hirschovits-Gerz B, Valkonen M (2019). The number and localization of CD68+ and CD163+ macrophages in different stages of cutaneous melanoma. Melanoma Res..

[10] Aras S, Zaidi MR (2017). TAMeless traitors: macrophages in cancer progression and metastasis. Br. J. Cancer.

[11] Ruffell B, Chang-Strachan D, Chan V, Rosenbusch A, Ho CM, Pryer N (2014). Macrophage IL-10 blocks CD8+ T cell-dependent responses to chemotherapy by suppressing IL-12 expression in intratumoral dendritic cells. Cancer Cell.

[12] Genin M, Clement F, Fattaccioli A, Raes M, Michiels C (2015). M1 and M2 macrophages derived from THP-1 cells differentially modulate the response of cancer cells to etoposide. BMC Cancer.

[13] Rolny C, Mazzone M, Tugues S, Laoui D, Johansson I, Coulon C (2011). HRG inhibits tumor growth and metastasis by inducing macrophage polarization and vessel normalization through downregulation of PlGF. Cancer Cell.

[14] Wang SC, Yu CF, Hong JH, Tsai CS, Chiang CS (2013). Radiation therapy-induced tumor invasiveness is associated with SDF-1-regulated macrophage mobilization and vasculogenesis. PLoS ONE.

[15] de Groot AE, Pienta KJ (2018). Epigenetic control of macrophage polarization: implications for targeting tumor-associated macrophages. Oncotarget.

[16] Steidl C, Lee T, Shah SP, Farinha P, Han G, Nayar T (2010). Tumor-associated macrophages and survival in classic Hodgkin’s lymphoma. N. Engl. J. Med..

[17] Hamann J, Koning N, Pouwels W, Ulfman LH, van Eijk M, Stacey M (2007). EMR1, the human homolog of F4/80, is an eosinophil-specific receptor. Eur. J. Immunol..

[18] Lyons YA, Pradeep S, Wu SY, Haemmerle M, Hansen JM, Wagner MJ (2017). Macrophage depletion through colony stimulating factor 1 receptor pathway blockade overcomes adaptive resistance to anti-VEGF therapy. Oncotarget.

[19] Ngiow SF, Meeth KM, Stannard K, Barkauskas DS, Bollag G, Bosenberg M (2016). Co-inhibition of colony stimulating factor-1 receptor and BRAF oncogene in mouse models of BRAFV600E melanoma. Oncoimmunology.

[20] Holt DJ, Grainger DW (2012). Senescence and quiescence induced compromised function in cultured macrophages. Biomaterials.

[21] Kelley JL, Rozek MM, Suenram CA, Schwartz CJ (1987). Activation of human blood monocytes by adherence to tissue culture plastic surfaces. Exp. Mol. Pathol..

[22] Newman SL, Tucci MA (1990). Regulation of human monocyte/macrophage function by extracellular matrix. Adherence of monocytes to collagen matrices enhances phagocytosis of opsonized bacteria by activation of complement receptors and enhancement of Fc receptor function. J. Clin. Invest..

[23] Sun S, Pan X, Zhao L, Zhou J, Wang H, Sun Y (2016). The expression and relationship of CD68-tumor-associated macrophages and microvascular density with the prognosis of patients with laryngeal squamous cell carcinoma. Clin. Exp. Otorhinolaryngol..

[24] Zhang M, He Y, Sun X, Li Q, Wang W, Zhao A (2014). A high M1/M2 ratio of tumor-associated macrophages is associated with extended survival in ovarian cancer patients. J. Ovarian Res..

[25] Yuan A, Hsiao Y-J, Chen H-Y, Chen H-W, Ho C-C, Chen Y-Y (2015). Opposite effects of M1 and M2 macrophage subtypes on lung cancer progression. Sci. Rep..

[26] Gordon S, Plüddemann A, Martinez Estrada F (2014). Macrophage heterogeneity in tissues: phenotypic diversity and functions. Immunol. Rev..

[27] Breslow AThickness (1970). cross-sectional areas and depth of invasion in the prognosis of cutaneous melanoma. Ann. Surg..

[28] Torisu H, Ono M, Kiryu H, Furue M, Ohmoto Y, Nakayama J (2000). Macrophage infiltration correlates with tumor stage and angiogenesis in human malignant melanoma: possible involvement of TNFalpha and IL-1alpha. Int. J. Cancer.

[29] Lee WJ, Lee MH, Kim HT, Won CH, Lee MW, Choi JH (2019). Prognostic significance of CD163 expression and its correlation with cyclooxygenase-2 and vascular endothelial growth factor expression in cutaneous melanoma. Melanoma Res..

[30] Mantovani A, Locati M (2013). Tumor-associated macrophages as a paradigm of macrophage plasticity, diversity, and polarization: lessons and open questions. Arterioscler Thromb. Vasc. Biol..

[31] Bardi GT, Smith MA, Hood JL (2018). Melanoma exosomes promote mixed M1 and M2 macrophage polarization. Cytokine.

[32] Najafi M, Hashemi Goradel N, Farhood B, Salehi E, Nashtaei MS, Khanlarkhani N (2019). Macrophage polarity in cancer: a review. J. Cell. Biochem..

[33] Nywening TM, Wang-Gillam A, Sanford DE, Belt BA, Panni RZ, Cusworth BM (2016). Targeting tumour-associated macrophages with CCR2 inhibition in combination with FOLFIRINOX in patients with borderline resectable and locally advanced pancreatic cancer: a single-centre, open-label, dose-finding, non-randomised, phase 1b trial. Lancet Oncol..

[34] Sanford DE, Belt BA, Panni RZ, Mayer A, Deshpande AD, Carpenter D (2013). Inflammatory monocyte mobilization decreases patient survival in pancreatic cancer: a role for targeting the CCL2/CCR2 axis. Clin. Cancer Res..

[35] Grossman JG, Nywening TM, Belt BA, Panni RZ, Krasnick BA, DeNardo DG (2018). Recruitment of CCR2(+) tumor associated macrophage to sites of liver metastasis confers a poor prognosis in human colorectal cancer. Oncoimmunology.

[36] Mantovani A, Sozzani S, Locati M, Allavena P, Sica A (2002). Macrophage polarization: tumor-associated macrophages as a paradigm for polarized M2 mononuclear phagocytes. Trends Immunol..

[37] Jeong H, Kim S (2019). Tumor-associated macrophages enhance tumor hypoxia and aerobic glycolysis. Cancer Res..

[38] Comunanza V, Corà D, Orso F, Consonni FM, Middonti E, Di Nicolantonio F (2017). VEGF blockade enhances the antitumor effect of BRAFV600E inhibition. EMBO Mol. Med..

[39] Romano E, Kusio-Kobialka M, Foukas PG, Baumgaertner P, Meyer C, Ballabeni P (2015). Ipilimumab-dependent cell-mediated cytotoxicity of regulatory T cells ex vivo by nonclassical monocytes in melanoma patients. Proc. Natl Acad. Sci. USA.

[40] Wolchok JD, Chiarion-Sileni V, Gonzalez R, Rutkowski P, Grob JJ, Cowey CL (2017). Overall survival with combined nivolumab and ipilimumab in advanced melanoma. N. Engl. J. Med..

[41] Engeland K (2018). Cell cycle arrest through indirect transcriptional repression by p53: I have a DREAM. Cell Death Differ..

[42] Gottfried E, Kunz-Schughart LA, Weber A, Rehli M, Peuker A, Muller A (2008). Expression of CD68 in non-myeloid cell types. Scand. J. Immunol..

[43] Greaves DR, Quinn CM, Seldin MF, Gordon S (1998). Functional comparison of the murine macrosialin and human CD68 promoters in macrophage and nonmacrophage cell lines. Genomics.

[44] Pillai MM, Hayes B, Torok-Storb B (2009). Inducible transgenes under the control of the hCD68 promoter identifies mouse macrophages with a distribution that differs from the F4/80- and CSF-1R-expressing populations. Exp. Hematol..

