# Supplementary material for: Differential association of CD68+ and CD163+ macrophages with macrophage enzymes, whole tumour gene expression and overall survival in advanced melanoma
Source: Br J Cancer. 2020 Aug 26;123(10):1553–61. doi: 10.1038/s41416-020-01037-7 (PMC7653046; doi:10.1038/s41416-020-01037-7)
Supplement: Supplementary file 1 — Supplementary material [file 41416_2020_1037_MOESM1_ESM.pdf]

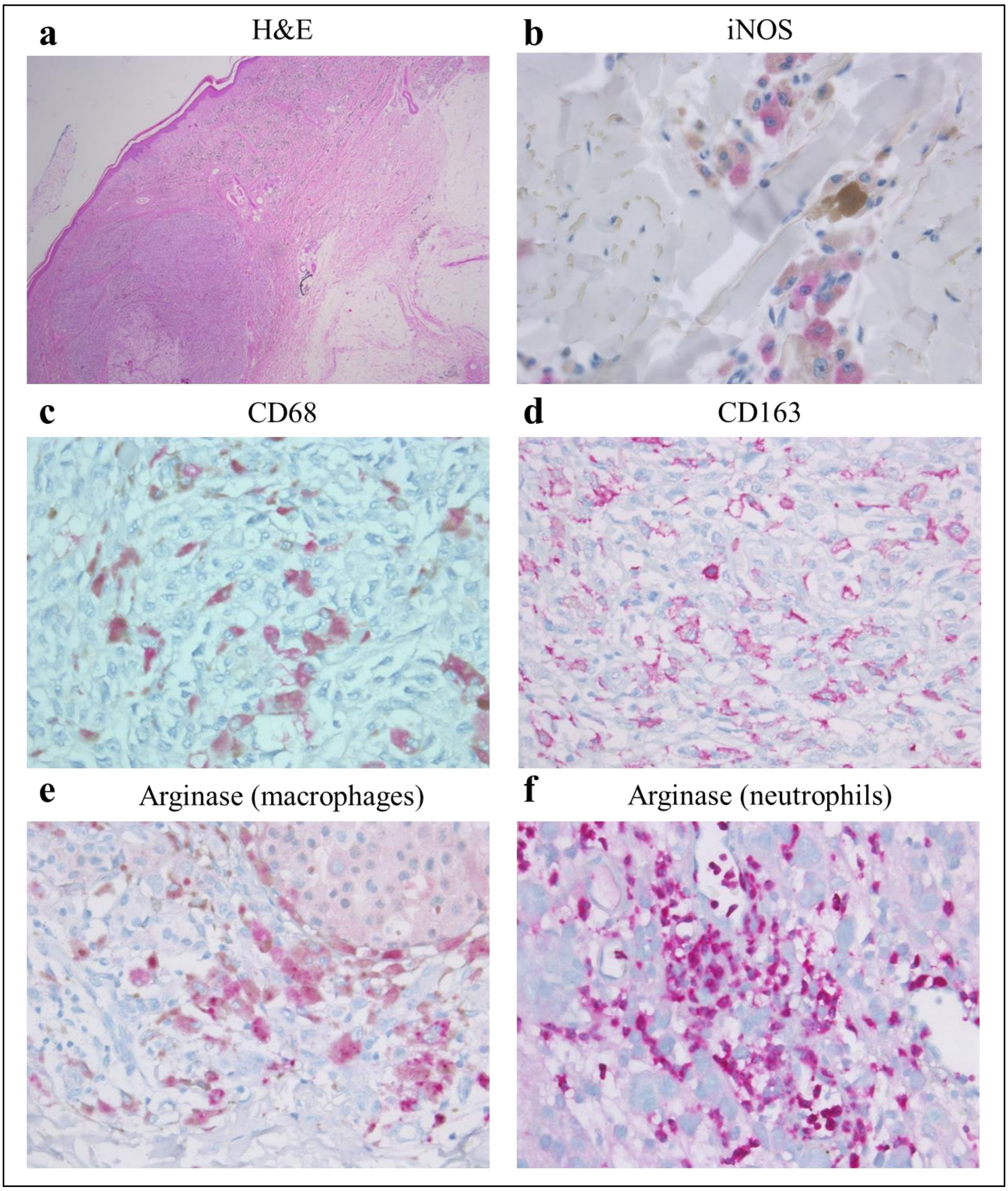

Figure 1 Staining of primary melanoma tumours

FFPE tumours were cut and stained by H&E (a), along with immunohistochemical staining of iNOS (b), CD68 (c), CD163 (d) and arginase (e-f). Immunohistochemical staining was performed using an alkaline phosphatase coupled to Fast-Red to produce a red/pink reaction. Images are shown at 40x (a), 400x (b) or 100x (c-f) magnification.

Table 1 Pathological features of the tumours stained by immunohistochemistry

Patient Characteristics			
**Characteristic**		**No.**	**%**
Sex	Male	35	(61)
	Female	22	(39)
Age	Median	64 years	
	Range	18-85 years	
Subtype	Superficial spreading	20	(34)
	Nodular	19	(32)
	Acral lentiginous	7	(12)
	Lentigo maligna	6	(10)
	Desmoplastic	1	(2)
	Unknown	5	(8)
Primary location	Acral	11	(19)
	Head and neck	10	(18)
	Lower limb	15	(26)
	Upper limb	12	(21)
	Trunk	9	(26)
Breslow depth	<1mm	5	(8)
	1.01-2mm	7	(12)
	2.01-4mm	13	(22)
	>4.01mm	32	(54)
Stage	pT1a	2	(4)
	pT1b	3	(5)
	pT2a	7	(12)
	pT3a	8	(14)
	pT3b	4	(7)
	pT4a	5	(9)
	pT4b	28	(49)
Mitotic count	Median	6.5mm	
	Average	10.17mm	
	Range	0-51mm	
BRAF status	Positive	24	(42)
	Negative	33	(58)
Ulceration	Yes	33	(49)
	No	34	(51)
Tumour infiltrating lymphocytes	Absent	14	(24)
	Non-brisk	36	(63)
	Brisk	3	(5)
	Variable	2	(4)
	Unknown	2	(4)

Random primary melanoma tumours which had undergone routine testing were selected for analysis by immunohistochemistry. Above is the cohort descriptives characterizing both the patient and the melanoma.

Table 2 Pathological features of the tumours selected for gene expression

Patient Characteristics

**Characteristic**		**No.**	**%**
Sex	Male	13	(65)
	Female	7	(35)
Age	Median	70.5 years	
	Range	26-85 years	
Subtype	Superficial spreading	5	(25)
	Nodular	11	(55)
	Acral lentiginous	2	(10)
	Unknown	2	(10)
Primary location	Acral	4	(20)
	Head and neck	3	(15)
	Lower limb	5	(25)
	Upper limb	5	(25)
	Trunk	3	(15)
Breslow depth	<1mm	0	(0)
	1.01-2mm	1	(5)
	2.01-4mm	2	(10)
	>4.01mm	17	(85)
Stage	pT2a	1	(5)
	pT3a	3	(15)
	pT4b	16	(80)
Mitotic count	Median	12mm	
	Average	11.89mm	
	Range	0-30mm	
BRAF status	Positive	6	(30)
	Negative	14	(70)
Ulceration	Yes	15	(75)
	No	5	(25)
Tumour infiltrating lymphocytes	Absent	3	(15)
	Non-brisk	13	(65)
	Brisk	3	(15)
	Unknown	1	(5)

Random primary melanoma tumours which had undergone routine testing were selected for analysis by immunohistochemistry. 24 of these tumours were selected for gene expression analysis based on tumour tissue availability within the FFPE block. 4 failed internal quality control and were omitted from analysis. Above is the cohort descriptives characterizing both the patient and the melanoma of tumours which were successfully analysed for gene expression and presented in chapter 3.

Table 3 Association of pathological tumour features with CD68^{+} and CD163^{+} macrophage infiltration

	**CD68**		**CD163**	
	**Peritumoural**	**Intratumoural**	**Peritumoural**	**Intratumoural**
Age	0.4	0.19	0.233 (0.162)	0.502 (0.092)
Gender	0.425	0.834	0.498	0.78
Breslow depth	**0.004**	0.5	0.937 (0.011)	**0.0191 (0.315)**
Stage	0.28	0.72	0.62	0.213
BRAF status	**0.027**	**0.001**	0.443	0.726
Mitotic count (mm)	**0.01**	0.13	0.908 (-0.017)	0.501 (-0.017)
Ulceration (mm)	0.12	0.36	0.074 (0.356)	0.208 (0.255)
Ulceration (Yes/No)	**0.01**	0.224	0.549	0.683
TIL distribution	0.683	0.455	0.166	0.353

The association of the number of CD68+ and CD163+ cells with patient and tumour characteristics was determined using Wilcoxon Rank Sum test and Spearman Correlations. The significance value for each relationship is shown above, and where relevant the correlation coefficient is also shown. Data is presented as significance values (correlation coefficients), n=57. With significant correlations in bold text.

Table 4 Association of pathological tumour features with iNOS^{+} and Arginase^{+} cell density, presented as significance values

	**Arginase**		**iNOS**	
	**Peritumoural**	**Intratumoural**	**Peritumoural**	**Intratumoural**
**Age**	0.242	0.861	0.28	0.17
**Gender**	0.921	0.721	0.62	0.69
**Breslow depth**	0.947	0.671	0.4	0.2
**Stage**	0.894	0.482	0.09	0.16
**BRAF status**	0.416	0.693	**0.0002**	**0.0002**
**Mitotic count (mm)**	0.492	0.084	0.28	0.35
**Ulceration (mm)**	**0.00015**	0.416	0.15	0.67
**Ulceration (Yes/No)**	0.795	0.33	**0.008**	**0.005**
**TIL distribution**	0.278	0.582	0.96	0.77

The association of the number of arginase^{+} and iNOS^{+} cells with patient and tumour characteristics was determined using Wilcoxon Rank Sum test and Spearman Correlations. Data is presented as significance values, n=57. With significant correlations in bold text.
